# Predicting odor from vibrational spectra: a data-driven approach

**DOI:** 10.1038/s41598-024-70696-w

**Published:** 2024-09-02

**Authors:** Durgesh Ameta, Laxmidhar Behera, Aniruddha Chakraborty, Tushar Sandhan

**Affiliations:** 1 Indian Knowledge System and Mental Health Applications Centre, Indian Institute of Technology, Mandi, 175005 India; 2 Department of Electrical Engineering, Indian Institute of Technology, Kanpur, 208016 India; 3 School of Basic Sciences, Indian Institute of Technology, Mandi, 175001 India; 4 Indian Knowledge System Centre, ISS, Delhi, 110065 India

**Keywords:** Odor prediction, Vibrational Spectra, Explainable AI, Cheminformatics, Computational chemistry

## Abstract

This study investigates olfaction, a complex and not well-understood sensory modality. The chemical mechanism behind smell can be described by so far proposed two theories: vibrational and docking theories. The vibrational theory has been gaining acceptance lately but needs more extensive validation. To fill this gap for the first time, we, with the help of data-driven classification, clustering, and Explainable AI techniques, systematically analyze a large dataset of vibrational spectra (VS) of 3018 molecules obtained from the atomistic simulation. The study utlizes image representations of VS using Gramian Angular Fields and Markov Transition Fields, allowing computer vision techniques to be applied for better feature extraction and improved odor classification. Furthermore, we fuse the PCA-reduced fingerprint features with image features, which show additional improvement in classification results. We use two clustering methods, agglomerative hierarchical (AHC) and k-means, on dimensionality reduced (UMAP, MDS, t-SNE, and PCA) VS and image features, which shed further insight into the connections between molecular structure, VS, and odor. Additionally, we contrast our method with an earlier work that employed traditional machine learning on fingerprint features for the same dataset, and demonstrate that even with a representative subset of 3018 molecules, our deep learning model outperforms previous results. This comprehensive and systematic analysis highlights the potential of deep learning in furthering the field of olfactory research while confirming the vibrational theory of olfaction.

## Introduction

Scientifically, the sense of smell is intriguing due to its complexity, close connection to memory and emotions, and the ongoing discourse surrounding its underlying mechanisms. Within the olfactory epithelium, spanning a 3.7 cm zone in the upper nasal passages, olfactory receptor neurons interact with volatile substances, giving rise to the perception of odors^1^. As established through the Nobel Prize-winning work of Linda Buck and Richard Axel, olfactory receptors (ORs) belong to the G-Protein Coupled Receptor class^2^. The molecular characteristics of odorants determine their affinity for multiple ORs and vice versa. An action potential is generated by structural and electrochemical changes initiated by the binding of odorant and receptor, transmitting olfactory information to the brain. The present-day investigations are focused on understanding the mechanism that underlies the binding and triggering described, which is still not clearly understood^3^.

The chemical mechanism behind smell can be described by so far proposed two theories: vibrational and docking theories. According to Dyson^4^, who first proposed vibrational theory, the olfactory receptors detect the localized vibrations of smell molecules, much like a chemical spectroscope. Another subsequent and more widely accepted Shape Theory posits that following the binding of odorants to receptors, a conformational shift occurs in the receptors, turning them from an inactive to an active state, called docking or lock-and-key^5^. Reviving the Vibration Theory, Turin proposed Inelastic Electron Tunneling Spectroscopy (IETS) as a means of detecting vibrational energy of odorant^6^. Consequently, olfaction is now recognized as an exemplary system within the emerging domain of quantum biology^7^. Despite the significant disagreement around the Vibration Theory^8,9^, certain research indicates that molecular vibrations may contribute to the sense of smell^10–12^. The swipe card mechanism also suggests that molecular vibration energy is important in addition to docking^13^.

Our investigation delves into the vibrational theory, proposing a strong correlation between a molecule’s olfactory characteristics and its vibrational frequency in the infrared spectrum. The field of predicting molecular properties is changing due to the rise of data-driven and machine-learning methodologies^14–19^, which provide significant improvements over conventional statistical methods. This paper explores this new wave of approaches, concentrating on the prediction of molecule odor from vibrational frequencies. The emphasis lies on multi-label classification, employing advanced deep-learning methodologies.

This paper’s key contributions are (1) We assembled a large and novel dataset of VS; (2) systematic analysis of VS using data-driven clustering, deep learning classification methods, and Explainable AI that reveal a relationship between VS, odor, and shape; (3) unimodal and multimodal deep learning models for improved odor classification.

This paper is structured as follows: The first section details the datasets (odor and VS) and explains the featurization of the datasets into fingerprints, gaussian smoothed spectra and image representation; then, it describes models and methods for classification (cost-sensitive deep learning models), dimensionality reduction (UMAP, MDS, t-SNE, PCA), clustering(AHC, k-mean) and saliency analysis (permutation analysis, GradCam++). The following section details the results of classification and clustering, followed by an analysis of constituent molecules within each cluster and a discussion on important features obtained by saliency analysis. Finally, conclusions are presented in the last section.

## Materials and methods

This section starts with describing the datasets and featurizing them, following that, classification, clustering, and saliency analysis methods are described.

### Dataset

The characterization of odorants involves the consideration of various qualitative descriptors, including hedonic attributes. Of the various odorant datasets with their odor labels available, we used a combined dataset from the training materials provided by Firmenich during the “Learning to Smell Challenge” with the Leffingwell PMP 2001^20^. This combined dataset has 7,374 molecules with significant structural diversity and 109 distinct odor classes. We also used VS of odorant molecules, as receptors detect vibrational energy reflected in VS by the mechanism of IETS, as mentioned previously.

#### Integrated dataset (IGD)

**Fig. 1:**
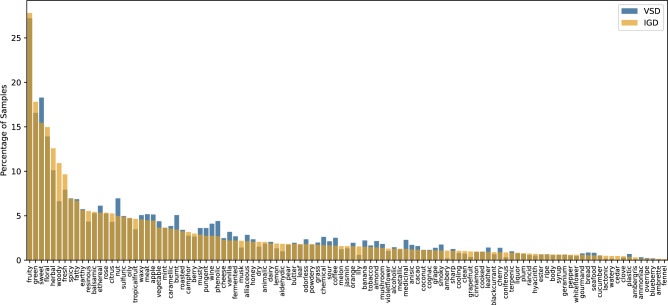
There is a significant imbalance in associated samples of odor labels, with the “fruity” label appearing around 27% of the time, whereas the occurrence of “fennel” is less than 1% in the dataset.

Saini et al. compiled a dataset by combining two distinct sets of expertly labeled odor datasets^20^. These sets originated from the training materials provided by Firmenich during the “Learning to Smell Challenge” and Leffingwell PMP 2001. The resulting dataset, after merging and preprocessing, comprised 7374 molecules distributed across 109 unique odor classes, each with different sample counts, as illustrated in Fig. 1. The complete list of all 7374 odorant molecules can be found in Supplementary file 1.

#### Subset of integrated dataset (Subset-IGD)

The CAS Registry number or International Chemical Identifier (InChI) Code was generated for all IGD molecules by utilizing their SMILE code^21,22^. The conversion from SMILE to CAS or InChI was done using the NistChemPy Python library^23^. Subsequently, the Vibrational Spectra (VS) corresponding to the obtained CAS and InChI were acquired from the Chemistry Webbook hosted by the National Institute of Standards and Technology (NIST)^24^. Vibrational frequencies and intensities of all these molecules were determined with the B3LYP/6-31G* level of theory, a type of density functional theory (DFT), after optimizing the molecular structures for minimum energy. Gaussian 09 suite^25^ was used for DFT calculations. Although IGD had 7374 molecules, the molecules without VS were eliminated and 3018 molecules were left in Subset-IGD having 109 unique odors. Consequently, Subset-IGD emerged as a representative sub-dataset derived from IGD. Figure 1 shows the percentage of samples associated with odor classes in IGD and Subset-IGD, visually indicating that Subset-IGD is a representative sub-dataset of IGD. This is confirmed by the Kolmogorov-Smirnov (K-S) test^26^, which yielded a test statistic (D) of 0.0734 and a p-value of 0.93, signifying Subset-IGD to be a representative sub-dataset of IGD. All 3018 odorant molecules are listed in Supplementary file 1.

### Featurising molecules

We featurized molecules and their VS to get a meaningful numerical representation for training our classification models. Molecules were featurized using traditional daylight fingerprinting as they gave the best result on the used dataset in the previous studies. We experimented with the Morgan fingerprint, too, also known as the extended-connectivity fingerprint (ECFP4) having bit length of 2048 bits and the fragment radius was set to 2 (diameter 4)^27^, RDKit was used to generate these features but they performed poorly compared to Daylight fingerprints^28^; classification results of Morgan Fingerprint are detailed in supplementary file 5. Gaussian smoothing was performed on VS, followed by downsampling to get features. Smoothing broadens peaks in VS, and downsampling reduces the input dimension to the model, improving model performance^29^. Three channel images of VS were generated using GASF, GADF and MTF transformation; this allows the utilisation of advanced pattern recognition techniques, improving classification accuracy^30^. Hence, we had three types of features with which we carried out further analysis: Daylight Fingerprint Features (DFF), Gaussian Smoothed Dimensionally reduced features (GS_VS), Images presentation of VS and ResNet50 features (VS_IMG).

#### Gaussian smoothed dimensionally reduced features for VS (GS_VS)

For each spectrum, a set of wavenumbers (vwns) associated with a specific molecule is projected onto a linear bounded frequency scale (BFS), usually ranging from 1 to 4000 cm^−1^. This range is chosen to encompass all essential vibrational normal modes for subsequent analysis. Then, Gaussian smoothing is applied to the spectra. The BFS is normalized and sampled at fixed increments of L cm^−1^ for dimensionality reduction. The resulting spectra are utilized as features for the deep learning model, referred to henceforth as GS_VS^29^. The equation for a Gaussian kernel is:

1 $$\begin{aligned} K_{\text {gaussian}} = \frac{1}{\sigma \sqrt{2\pi }} \exp \left( \frac{(x - \bar{x})^2}{2\sigma ^2}\right) \end{aligned}$$

**Fig. 2:**
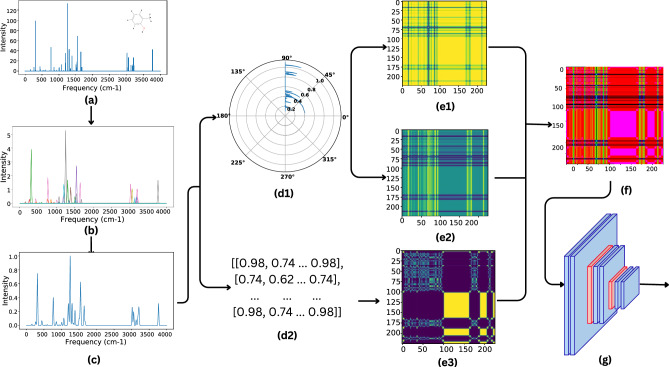
Overview of feature generation from VS for molecule 2-methyl-phenol (shown in inset). (**a**) Projected VS onto a BFS (**b**) spectra obtained after Gaussian smoothing (**c**) normalised spectra with BFS sampled at 5 cm^−1^. Obtained spectra in (**c**) is further processed to generate image features using GAF and MTF (**d1**) polar coordinate mapping (**d2**) Markov transition matrix (**e1**) Gramian angular summation field (GASF) a 224X224X1 image (**e2**) Gramian angular difference field (GADF) a 224X224X1 image (**e3**) Markov transition field (MTF) a 224X224X1 image (**f**) GASF, GADF and MTF are overlayed to obtain a 224X224X3 image for each VS (**g**) pre-trained ResNet50 model is used to get flattened feature VS_IMG.

A sigma value of 10 cm^−1^ and L set to 5 cm^−1^ were employed, resulting 800 (4000/L) descriptor variables. The smoothing procedure applies a smearing function that broadens the vibrational peaks, which allows for comparing different compounds with slightly different frequencies. Figure 2 shows an overview of this process. Turner et al. introduced EVA descriptor for spectra, which has been validated for use in QSAR studies^31^. Proposed GS_VS performs equally well as the EVA descriptor, but when 3-channel images are generated for the EVA descriptor, the model’s performance on them is significantly reduced. A possible reason for this poor performance could be that the intensity of peaks is not considered while generating them. Hence, we use GS_VS for our research.

#### Fingerprint-based features (DFF)

To generate features for our deep learning model, we used a traditional featurization technique: daylight fingerprinting using the Rdkit. These Daylight fingerprint features (DFF) were generated from the SMILE representation of the molecules. These 1024 binary fingerprints capture the presence or absence of specific chemical substructures within a molecule.

#### Images presentation of VS and ResNet50 features (VS_IMG)

The obtained GS_VS is encoded into images using two frameworks, Markov Transition Field (MTF) and Gramian Angular field (GAF)^30^. These methods aid in transforming the time series into an image format, enhancing the classification process. Three images Gramian angular summation field (GASF) a 224 × 224 × 1 image, Gramian angular difference field (GADF) a 224 × 224 × 1 image, and Markov transition field (MTF) a 224X224X1 image obtained from GS_VS are overlayed to obtain a 224X224X3 image for each VS. We use pre-trained ResNet50 network (on ImageNet) to obtain the flattened features (VS_IMG) for these 3-channel images^32^. This is achieved with the help of both Python/keras.applications.resnet50 and Python/keras models. Figure 2 shows an overview of the process of converting GS_VS to VS_IMG.

#### Gramian angular field (GAF)

To create a 2-D image, the first step is to transform the normalized time series data into polar coordinates. While mapping to the polar coordinate system, the normalized time series value is represented as the angular cosine, and the time stamps are illustrated as the radius in the polar coordinate system. For a time series $Y = \{y_1, y_2, \ldots , y_n\}$, the angle ($\theta _i$) and radius ($r_i$) are computed using equations 2 and 3:

2 $$\begin{aligned} \theta _{\text {i}} = \cos ^{-1}(\overline{Y}_{\text {i}}); \quad \text {where},\ 0 \le \theta _i \le \frac{\pi }{2} \end{aligned}$$

3 $$\begin{aligned} r_{\text {i}} = \frac{t_{\text {i}}}{N} \end{aligned}$$

Where $\overline{Y}_{\text {i}}$ is normalized time series, $N$ and $t_i$ are the constant value and time stamp, respectively, that divide the polar coordinate’s time span into equal portions. When transitioning a time series from the Cartesian coordinate system to the polar coordinate system, the GAF transformation exhibits the property of bijectiveness, generating a unique value in the polar system and a distinct inverse value. Unlike the Cartesian coordinate system, the absolute temporal relationship is preserved within the polar coordinate system. Consequently, a Gramian matrix is constructed, where each matrix element corresponds to the trigonometric function of the sum or difference across different time intervals. By employing the following equations, GASF and GADF can be derived:

$$\begin{aligned} GASF = \left[ \begin{array}{ccc} \cos (\theta _{1}+\theta _{1}) &{} \cdots &{} \cos (\theta _{1}+\theta _{n}) \\ \vdots &{} \ddots &{} \vdots \\ \cos (\theta _{n}+\theta _{1}) &{} \cdots &{} \cos (\theta _{n}+\theta _{n}) \end{array} \right] \\ GADF = \left[ \begin{array}{ccc} \sin (\theta _{1}-\theta _{1}) &{} \cdots &{} \sin (\theta _{1}-\theta _{n}) \\ \vdots &{} \ddots &{} \vdots \\ \sin (\theta _{n}-\theta _{1}) &{} \cdots &{} \sin (\theta _{n}-\theta _{n}) \end{array} \right] \end{aligned}$$

#### Markov transition field (MTF)

Dividing a data series $Y = \{y_1, y_2, \ldots , y_n\}$ evenly into *Q* bins, each $y_i$ is associated with a bin $q_j$ ($j \in [1, Q]$). Hence, by using a first-order Markov chain, a Markov matrix *Z* of size $Q \times Q$ can be built. It makes the following expression: $z_{\text {ij}}=p\{ y_{\text {t}}\in q_{\text {i}}|y_{\text {t-1}} \in q_{\text {j}}\}$

$$\begin{aligned} Z = \left[ \begin{array}{ccc} z_{\text {11}} &{} z_{\text {12}} \cdots &{} z_{\text {1Q}}\\ z_{\text {21}} &{} z_{\text {22}} \cdots &{} z_{\text {2Q}}\\ \vdots &{} \ddots &{} \vdots \\ z_{\text {Q1}} &{} z_{\text {Q2}} \cdots &{} z_{\text {QQ}}\\ \end{array} \right] \\ \end{aligned}$$

The likelihood of the point *y*, presently situated in bin $q_i$, transitioning to bin $q_j$ in the subsequent time is represented by $z_{ij}$. Evidently, $z_{ij}$ fulfils the relation: $\sum _{j=1}^{Q} z_{ij} = 1$. Due to the memorylessness property of the Markov chain, the current elements in the matrix *Z* are independent of the previous element, leading to a notable loss of pertinent data. To address this constraint, the matrix *Z* is expanded to the Markov transform field *N* by introducing a time axis. The data point at time stamps *i* and *j* is associated with respective bins: $q_i$ and $q_j$, following the division of the dataset into *Q* bins along the time axis. The transition probability from $q_i$ to $q_j$ is denoted by $N_{ij}$ and expressed as follows:

$$\begin{aligned} N = \left[ \begin{array}{ccc} z_{\text {ij}} | x_{\text {1}} \in q_{\text {i}},x_{\text {1}} \in q_{\text {j}} &{} \cdots &{} z_{\text {ij}} | x_{\text {1}} \in q_{\text {i}},x_{\text {n}} \in q_{\text {j}}\\ z_{\text {ij}} | x_{\text {2}} \in q_{\text {i}},x_{\text {1}} \in q_{\text {j}} &{} \cdots &{} z_{\text {ij}} | x_{\text {2}} \in q_{\text {i}},x_{\text {n}} \in q_{\text {j}}\\ \vdots &{} \ddots &{} \vdots \\ z_{\text {ij}} | x_{\text {n}} \in q_{\text {i}},x_{\text {1}} \in q_{\text {j}} &{} \cdots &{} z_{\text {ij}} | x_{\text {n}} \in q_{\text {i}},x_{\text {n}} \in q_{\text {j}} \end{array} \right] \end{aligned}$$

### Data analysis

To perform a systematic analysis of VS both clustering and classification were performed, and also saliency analysis of the model was done to explain model predictions.

#### Classification models

The histogram plot in Fig. 1 indicates that fruity is the highest occurring label, and fennel/cedar has the lowest occurrence in our dataset. As apparent, the distribution has a heavy class imbalance and is highly skewed.

**Fig. 3:**
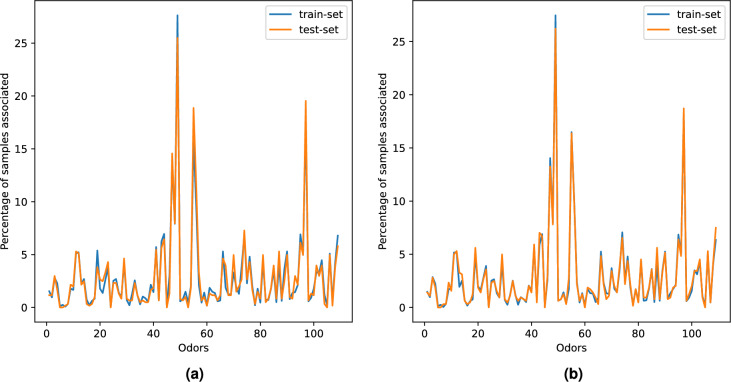
Data split into test and training sets using random and iterative sampling.

To ensure a general representation of data in both the training and test datasets, we opted for iterative stratified sampling instead of random sampling for the dataset split. This method was executed using the iterative_train_test_split class available in the scikit-multilearn library^33^. Choosing random sampling unintentionally led to the omission of minority odor descriptors, worsening the pre-existing label imbalance. Visualization in Fig. 3a and b illustrates the data split into test and training sets using random and iterative sampling, respectively. The line charts present a comparative analysis between random and stratified splits, where the non-overlap of the orange and blue line chart indicates the relative occurrence disparity of a specific label in the training and test sets. The findings highlight that stratified sampling yields a better representative test and training set split.

Given the considerable class imbalance in our dataset, we employed a cost-sensitive multilayer perceptron (CSMLP) for this study. The conventional multilayer perceptron (MLP) trained through the backpropagation of error algorithm assumes equal misclassification costs (false negative and false positive). However, recognizing that a false negative is more detrimental or costly than a false positive^34^, we introduced the CSMLP to address this issue. In the proposed CSMLP, we adapted the MLP’s loss function to accommodate class imbalance. To enhance the penalty for mistakes on minority class samples, we utilized a combination of weighted binary cross entropy (WBCEL) and focal loss. In WBCEL, assigned weights are proportional to the $\delta$ power of the inverse of class frequencies^35–37^, thereby imposing a higher penalty for errors on the minority class. Focal loss assigns a weight to each sample based on its difficulty, which is measured in terms of the loss incurred by the CSMLP on that particular sample^38^. Samples with higher losses are regarded as more challenging. The equations for Focal Loss (FL), Weighted Binary Cross-Entropy Loss (WBCEL), and Combined Loss (CL) are as follows:

4 $$\begin{aligned}{} & {} \text {FL} = -\frac{1}{N}\sum _{i=1}^{N}\sum _{j=1}^{C}((1 - y_{\text {pred,ij}})^\gamma \cdot y_{\text {true,ij}}\cdot \log (y_{\text {pred,ij}}+\varepsilon )+(y_{\text {pred,ij}})^\gamma \cdot (1 - y_{\text {true,ij}})\cdot \log (1 - y_{\text {pred,ij}}+\varepsilon )) \end{aligned}$$

5 $$\begin{aligned}{} & {} \text {WBCEL} = -\frac{1}{N}\sum _{i=1}^{N}\sum _{j=1}^{C}(w_{\text {pos,j}}^\delta \cdot y_{\text {true,ij}}\cdot \log (y_{\text {pred,ij}}+\varepsilon ) + w_{\text {neg,j}}^\delta \cdot (1 - y_{\text {true,ij}})\cdot \log (1 - y_{\text {pred,ij}}+\varepsilon )) \end{aligned}$$

6 $$\begin{aligned}{} & {} CL = WBCEL + \lambda *FL \end{aligned}$$

$\varvec{\lambda }$ is the hyper-parameter that controls the importance of each loss.

$\varvec{\gamma }$ is focusing paramerter in FL and $\delta$ is used for smoothing the effect of class weight.

**C** is number of classes and **N** is number of samples in a batch. ${\textbf {y}}_{{{\textbf {true,ij}}}}$ true label for jth class of ith example.

${\textbf {y}}_{{{\textbf {pred,ij}}}}$ predicted probability for jth class of ith example.$\varvec{\varepsilon }$ is a small constant to avoid numerical instability.

${\textbf {w}}_{{\textbf {pos,j}}}$ = (Number of -ve labels for class j)/(Total number of samples in dataset)

${\textbf {w}}_{{\textbf {neg,j}}}$ = (Number of +ve labels for class j)/(Total number of samples in dataset)

**Fig. 4:**
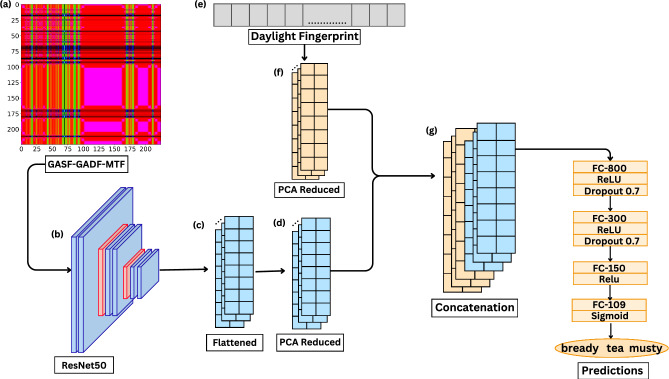
Diagram of fusion architecture that learns jointly from VS_IMG and DFF. (**a**) VS_IMG a 224X224X3 image obtained by overlaying GASF-GADF-MTF (**b,c**) ResNet50 pre-trained model is then used to extract features from VS_IMG (**d**) these flattened features are then dimensionally reduced to 3036 using PCA (**e**) DFF for same molecule obtained from RDKit (**f**) DFF are dimensionally reduced using PCA (**g**) PCA reduced features from (**d**), (**f**) are concatenated and later classified using CSMLP.

We first trained unimodal baseline models on PCA-reduced VS_IMG, DFF of Subset-IGD and IGD, and GS_VS features alone. We then developed multimodal fusion model that learn jointly from PCA-reduced VS_IMG and DFF. Figure 4 shows an overview of the fusion model.

All these models are CSMLP with fully connected layers, for activation function we have used Relu, and at the final layer, we used sigmoid. Models were trained with Adam^39^(keras-optimizer), and dropout was used as a regularizer to prevent overfitting. Along with CSMLP, we tried random resampling techniques: Multi-Label Random Under-Sampling (ML-RUS) and Multi-Label Random Over-Sampling (ML-ROS). However, we did not observe any significant improvement in results. We leave the detailed architecture of all models in the supplementary file 2 and results of resampling in the supplementary file 5.

#### Fusion model

**Fig. 5:**
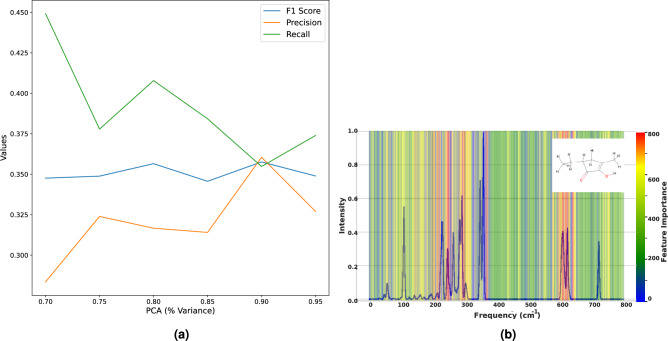
(**a**) Plot depicts the change in precision, recall and F1 score with respect to change in % variance of PCA-reduced DFF features. (**b**) Relevance produced by PFI methods, the contour maps represent the feature importance produced by the interpretability method, overlayed on spectra of 2-Cyclopenten-1-one, 2-hydroxy-5-ethyl-3-methyl (shown in inset). Red indicates important features, and it decreases from red to blue. The intensities of wavenumber 1005-1670 and 2985-3215 (range 201-334 and 597-643 in dimensionally reduced GS_VS) contributes most significantly.

Fusion model learns from PCA-reduced VS_IMG concatenated by PCA-reduced DFF to produce a final prediction. Figure 4 shows an overview of the fusion model^40^. We generated 6 different sets of PCA-reduced DFF, with percentage variance ranging from 70 to 95%, and when concatenated with PCA-reduced VS_IMG, we saw an increase in the F1 score with increased variance. Figure 5a shows value of F1 score for different values of variance.

### Dimensionality reduction and clustering

Features obtained from GS_VS and VS_IMG are high dimensional; reducing the dimensionality to 2D helps visualise compounds in a 2D space and observe their distribution. PCA, t-SNE, UMAP and MDS were used for dimensionality reduction, and then clustering was performed using AHC and k-mean clustering.

#### Dimensionality reduction

Four-dimensionality reduction techniques were applied on GS_VS and VS_IMG: PCA, t-SNE, UMAP and MDS. All were applied using Python 3.7.6 packages. Principal Component Analysis (PCA) is a multivariate analysis technique designed to extract essential information through an orthogonal transformation^41^. This transformation generates correlated variables known as principal components, which are new linearly independent variables capturing the key features of the original data. Multidimensional Scaling (MDS) functions as a technique for network localization, revealing the relationships, whether similarity or dissimilarity, among pairs of objects within a given dataset. This information is then translated into distances between points within a two-dimensional space^42^. The t-SNE method, an evolution of Stochastic Neighbor Embedding (SNE), gauges the similarities between pairs of high-dimensional data objects and their subsequent two-dimensional embedding. Utilizing gradient descent, t-SNE minimizes Kullback-Liebler divergence to generate a two-dimensional embedding^43^. We followed the protocol suggested by Arora et. al. for better t-SNE visualization^44^. UMAP, a recent dimension reduction technique, accurately captures non-linear structures in large datasets^45^. Based on algebraic topology and Riemannian geometry, UMAP constructs a manifold. Our visualization process entails a delicate balance between local and global information, a balance contingent on the careful selection of values for two key parameters: the “minimum distance” and “number of neighbors”. The “number of neighbors” parameter plays a pivotal role in balancing local and global structural aspects, while the “minimum distance” parameter governs the grouping of points within the low-dimensional representation. Following extensive testing and evaluation of various values, we settled on fixed values of 15 for the number of neighbors and 0.1 for the minimum distance.

#### Clustering

Agglomerative Hierarchical Clustering (AHC) and K-means were employed on the reduced dimensions derived from the four preceding techniques. The objective was to categorize similar odor molecules into distinct groups. Previous studies have undertaken clustering analyses based on fingerprint features of odor molecules^46^, yet none have specifically addressed the clustering of large VS datasets. The clustering process was executed using Python packages on the two-dimensional data to tackle challenges associated with high-dimensional clustering^47^. For Agglomerative Hierarchical Clustering (AHC), the Euclidean distance matrix (2x2) for each molecule was computed, employing the “ward.D2” aggregative criterion. This criterion aims to minimize the inertia within classes while maximizing the inertia between classes. The clustering process involved successively grouping the two closest classes until a complete clustering tree was obtained. While hierarchical clustering is known for its simplicity and versatility in handling various similarity or distance measures, the irreversible merging of clusters restricts the ability to correct erroneous decisions^48,49^. In the case of k-means clustering, various numbers of centroids corresponding to the desired cluster count were experimented. Points were assigned to their nearest cluster during each iteration, and the algorithm iteratively refined cluster centroids until no further changes occurred^48,50^. It’s worth noting that the k-means clustering algorithm is sensitive to outliers and may be less effective when dealing with clusters that deviate from hyper-spherical shapes.

To ascertain the most suitable number of clusters, we constructed an “elbow” curve illustrating intra-cluster variability against the number of clusters for each dimensional reduction technique. Following the establishment of clusters using either k-means or AHC, we examined the distribution of odor notes within these clusters. Additionally, we explored the chemical groups or functions of molecules present in different clusters.

### Feature Importance analysis

The explainability of the classification model is very important for developing vibration-based biomimetic sensors. We used two techniques for feature importance analysis: Permutation feature importance (PFI) and GradCam++^51,52^. Both are model-agnostic methods and do not require model retraining. PFI can capture variable interaction, too. GradCam++ provides visual explanations of CNN model predictions in terms of better object localization than the state-of-the-art.

#### Permutation feature importance (PFI)

For the model trained on GS_VS features we performed feature importance analysis using PFI. Let *f* is trained/tested uni-modal model on GS_VS and its error measure *L*(*y*, *f*). Then feature importance as the extent to which a feature $X_{i}$ affects *L*[*y*, *f*(*X*)], on its own and through its interactions with $X_{i}$. Permutation importance was first introduced by Breiman (Breiman, 2001)^53^, the present study uses its model-agnostic version later proposed by Fisher, Rudin, and Dominici (2018)^51^. Let $e_{orig}=L(y, f(X)))$ be error with original data, and $e_{perm}=L(f(y, X_{j,perm})))$ be error with *jth* feature permuted then feature importance $FI_j$ is given by equation: $FI_j = e_{\text {orig}} - e_{\text {perm}}$

#### GradCAM++

For the model trained on VS_IMG, we conducted saliency analysis employing GradCAM++. GradCAM++^52^ computes the second-order derivative of the predicted probability for the smell category concerning the final convolutional layer:

7 $$\begin{aligned} A_{c}(\textbf{x})=\sum _{i}\sum _{j}\alpha _{ij}^{c}\phi _{i}(\textrm{x};w_{i})\phi _{j}(\textbf{x};w_{j}) \end{aligned}$$

where $\phi _i$ and $\phi _j$ represent the activation maps of the *i*th and *j*th feature maps, and $w_i$ and $w_j$ denote the weights associated with the *i*th and *j*th feature maps. Additionally, $\alpha _{ij}^c$ represents the weight derived from the global average pooling of gradients across the spatial dimensions.

Subsequently, the gradient-weighted class activation maps undergo global average pooling, given by equation 8, Z represents the number of spatial locations in the feature map. Finally, the heatmap is generated, as given in equation 9:

8 $$\begin{aligned} \alpha _{c}^{\text {GradCAM}++}(\textbf{x})=\frac{1}{Z} \sum _{i} \sum _{j} L_{c}^{\text {GradCAM}++}(\textbf{x})_{i j} \end{aligned}$$

9 $$\begin{aligned} L_{c}^{\text {GradCAM}++}(\textbf{x})=\text {ReLU}\left( \sum _{k} \alpha _{k}^{c}(\textbf{x}) \phi _{k}(\textbf{x}; w_{k})\right) \end{aligned}$$

The resultant heatmap in GradCAM++ signifies the regions in VS_IMG that held the utmost significance for the smell category prediction. A higher value at a specific location indicates the increased importance of that location in the classification process.

## Results and discussion

In this section, we discuss results of classification, clustering and saliency analysis.

### Classification results

We trained five different classification models as described earlier; four of these were unimodal models trained on GS_VS, PCA-reduced VS_IMG, DFF for Subset-IGD and IGD and the last one is a multimodal model trained on PCA-reduced VS_IMG and DFF. Model performance was evaluated on micro-averaged scores. Numerous experiments were undertaken by adjusting hyperparameters to refine the model and achieve optimal results. All five deep models underwent end-to-end training for 100 epochs, with model weights being restored from the epoch with the highest F1 score. Overall, for DFF of IGD and Subset-IGD, CSMLP significantly outperformed all the models utilized by Saini et. al.^20^. A possible reason for this improvement could be a large number of free parameters of deep models. These parameters give them the flexibility to fit highly complex data that traditional machine learning models are too simple to fit. The model trained on VS_IMG outperformed the model trained on GS_VS; the results highlight the potential of deep learning in olfaction and can be exploited further. The fusion model outperformed the unimodal model trained on VS_IMG. This model was trained on PCA-reduced VS_IMG and DFF, indicating how the two features complement each other. The findings can be interpreted in two ways: firstly, as an affirmation of the swipe card mechanism for olfaction proposed by Brookes et al.^13^, indicating potential involvement of vibrational energy in addition to docking; and secondly, as an indication that vibrational spectra, when augmented with physicochemical features, offer enhanced attributes for QSAR studies. It is noteworthy that complex activation mechanisms, extending beyond docking, are observed in biological processes such as cancer immunology^54^. Additionally, in the realm of QSAR studies for biochemical molecules, as in contexts like drug design, it is a common practice to employ vibrational spectra as a proxy for structure^29^.

We try to concatenate different numbers of PCA-reduced DFF with the variance of the reduced features varying from 70% to 95% to the PCA-reduced VS_IMG, Fig. 5a shows results for different combinations. Table 1 shows the results of all the classification models.

**Table 1:** Results of CSMLP classification models.

Work	Dataset(Features)	Classifier	F1 Score	Precision	Recall
This work	Subset-IGD(PCA-Reduced VS_IMG)		0.3425	0.3074	0.386
	Subset-IGD(GS_VS)		0.3341	0.2995	0.377
	Subset-IGD(DFF)	CSMLP	0.4064	0.3632	0.4614
	Subset-IGD (PCA-Reduced VS_IMG concatenated to PCA-Reduced DFF)		0.3576	0.3547	0.3604
	IGD(DFF)		0.4083	0.3603	0.4710
Saini et al.^20^	IGD (DFF)	Random Forest	0.3221	0.3757	0.2819
		Binary Relevance	0.3523	0.3563	0.3483
		Classifier Chain	0.3267	0.3745	0.2930

### Clustering results

**Fig. 6:**
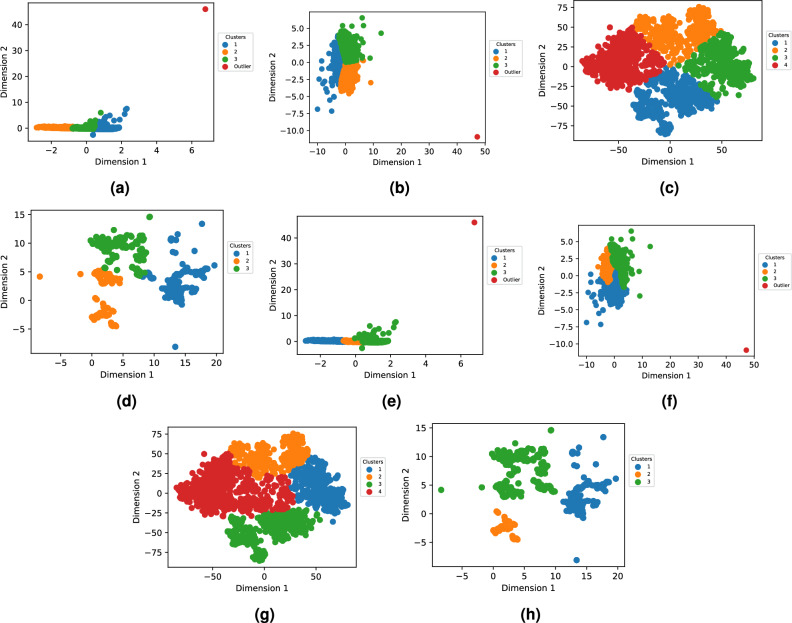
Visualization of the clusters in the two dimensional spaces obtained after dimension reduction of GS_VS using t-SNE, PCA, MDS, and UMAP. The data is color-coded based on the clusters generated by the AHC and k-means clustering conducted using the coordinates in the 2D spaces.

**Fig. 7:**
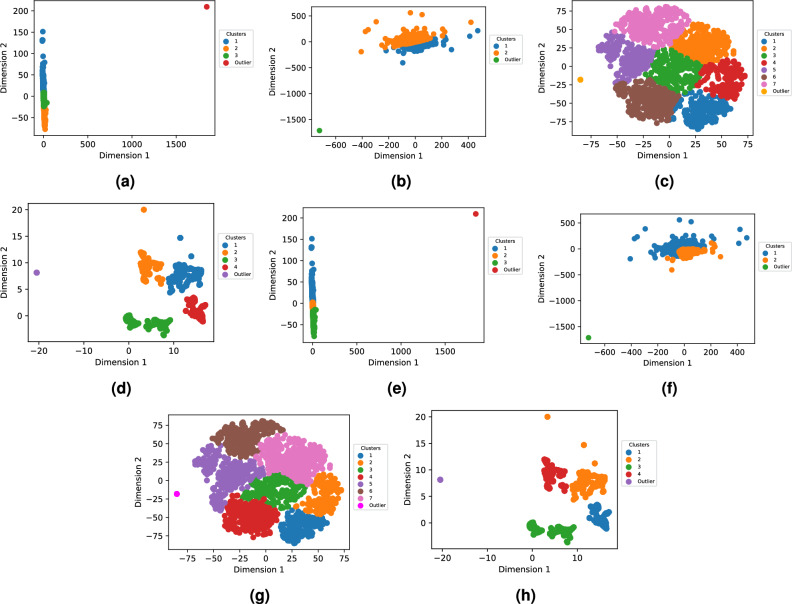
Visualization of the clusters in the 2-two dimensional spaces obtained after dimension reduction of VS_IMG using t-SNE, PCA, MDS and UMAP. The data is color-coded based on the clusters generated by the AHC and k-means clustering conducted using the coordinates in the 2D spaces.

Both VS_IMG and GS_VS were subjected to dimensionality reduction, yielding two-dimensional data through four distinct reduction techniques. Following this, the two clustering methods were employed on these 2D coordinates to group molecules based on their structural similarity. To identify the most suitable number of clusters, we generated an “elbow” curve depicting intra-cluster variability in relation to the number of clusters, details of the elbow curve can be found in Supplementary file 2. The positioning of olfactory compounds in the two-dimensional space, as defined by the computations from all techniques, is illustrated in Fig. 6 for GS_VS and Fig. 7 for VS_IMG.

Our investigation into cluster composition adopted two approaches: first, analyzing the frequencies of odor notes linked with the molecules, and second, determining the count of molecules featuring specific odor notes. Given the considerable variation in odor occurrences, spanning from 1 to 821, a direct comparison of occurrence numbers wouldn’t be reliable, particularly for less frequent odor notes.

10 $$\begin{aligned} \% \text {{odor notes}} = \%ON = \frac{{\text {{number of occurrences of an odor note in the cluster}}}}{{\text {{total number of occurrences of this odor}}}} \end{aligned}$$

11 $$\begin{aligned} \% \text {{odor samples}} = \%OM = \frac{{\text {{number of occurrences of an odor in the cluster}}}}{{\text {{number of elements molecules in this cluster}}}} \end{aligned}$$

As an illustration, employing the UMAP-kmeans methodology resulted in 1151 molecules being assigned to cluster C1. Among these, the odor note “green” occurred 500 times in the entire dataset, and within cluster C1, it appeared 201 times. Hence, approximately 40.2% of molecules associated with the “green” odor were consolidated in C1, forming 17.46% of the total molecules in this cluster.

$$\begin{aligned} {\textbf {\%ON ``green''}} = 201/500 = 40.2\% and {\textbf {\%OM ``green''}} = 201/1151 = 17.46\% \end{aligned}$$

**Fig. 8:**
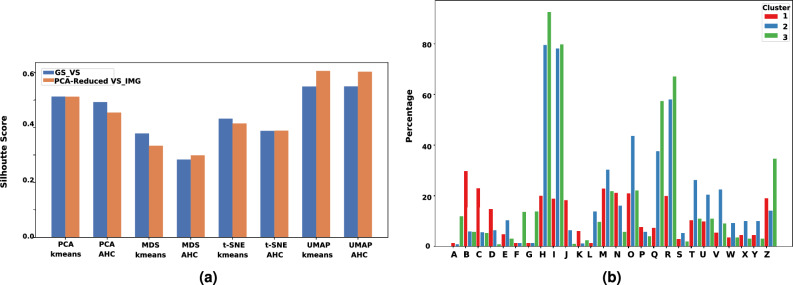
(**a**) Silhouette score for different dimensionality reduction techniques and clustering techniques for GS_VS and VS_IMG. (**b**) Histogram illustrating the distribution of chemical functional groups across clusters. (**A**) Alcohol(Al_coo) (**B**) Alcohol(Al_oh) (**C**) Alcholo(Al_oh_notert) (**D**) Aromatic Amine(Ar_n) (**E**) Aromatic Hydroxyl(Ar_oh) (**F**) Carboxylic Acid(Coo) (**G**) Carboxylic Acid(Coo2) (**H**) Carbonyl O (C_o) (**I**) Carbonyl O excluding Cooh (C_o_nocoo) (**J**) Tertiary amines (Nh0) (**K**) Thiol(Sh) (**L**) Aldehyde (**M**) Allylic_oxid (**N**) Aryl_methyl (**O**) Benzene (**P**) Bicyclic (**Q**) Ester (**R**) Ether (**S**) Furan (**T**) Ketone (**U**) Ketone_topliss (**V**) Methoxy (**W**) Para_hydroxylation (**X**) Phenol (**Y**) Phenol_noorthohbond (**Z**) Unbrch_alkane.

In assessing the efficacy of the employed techniques for distinguishing between odors, we calculated the silhouette score for each clustering technique across various features and dimensionality reduction methods^55^. As depicted in Fig. 8a, it is evident that UMAP exhibited the most pronounced discriminative capability.

For clarity, we concentrated on presenting the results related to UMAP for GS_VS only, while the clustering results from other methods and VS_IMG features are detailed in supplementary file 3.

The cluster dC1(UMAP k-means) was majorly constituted of ‘cofee’, ‘camphor’, ‘sulfuric’, ‘vegetable’, ‘earthy’, ‘meat’, ‘nut’, ‘oily’ and ‘wood’ with their %ON greater than 50%. In dc2 ‘vanilla’, ‘spicy’, and ‘sweet’ odors were present especially and in dc3 ‘sour’, ‘wine’, ‘apple’ were present predominantly. On the other hand, cluster hc1 especially had molecules with odors ‘vegetable’, ‘sulphuric’, ‘earthy’, ‘meat’, ‘nut’, hc2 had ‘coconut’, and ‘sour’. In hc3, the following odors were prominent ‘apple’, ‘balsamic’, ‘fruity’, ‘spicy’, ‘phenolic’, ‘and herbal’, they constituted more than 58% of molecules, although this cluster had molecules of all 109 odors present.

#### Chemical structures and functions of odorants

Using RdKit, we checked the presence or absence of 85 structures and functions with diverse characteristics within the molecules of clusters. The complete list of these 85 structures and functions can be found in Supplementary File 4. We focused on identifying chemical functional groups in a minimum of 5% of the molecules within any of the three clusters. For cluster d in Fig. 6, we get 26 such functional groups. Here, we limit the discussion to cluster d; for the rest of the clusters, the analysis can be found in supplementary file 4. As shown in Fig. 8b, carbonyl compounds were notably prevalent in clusters dC2. Moreover, the dC3 cluster had a significant presence of odorant molecules with carbonyl groups. Within these clusters, dC3 displayed a notably higher percentage (92.5%) of carbonyl compounds, primarily as ester functional group. In cluster dC2, molecules with benzene rings and ethers were prevalent, constituting 43% and 58% of the molecules, respectively. In contrast, molecules belonging to other clusters exhibited a scarcity of these chemical groups. Molecules with ether groups were particularly frequent in cluster dC3, comprising 45% of its composition.

### Feature importance analysis

#### Permutation feature importance (PFI)

Figure 5b illustrates the features identified as important through PFI analysis, superimposed on the spectra of 2-Cyclopenten-1-one, 2-hydroxy-5-ethyl-3-methyl. The figure highlights that the regions of interest for the modal are precisely those with the most significant physical information. Specifically, intensity values for wavenumber ranges 1005-1670 and 2985-3215 play a pivotal role in determining the distinctiveness of the modal.

#### GradCAM++

**Fig. 9:**
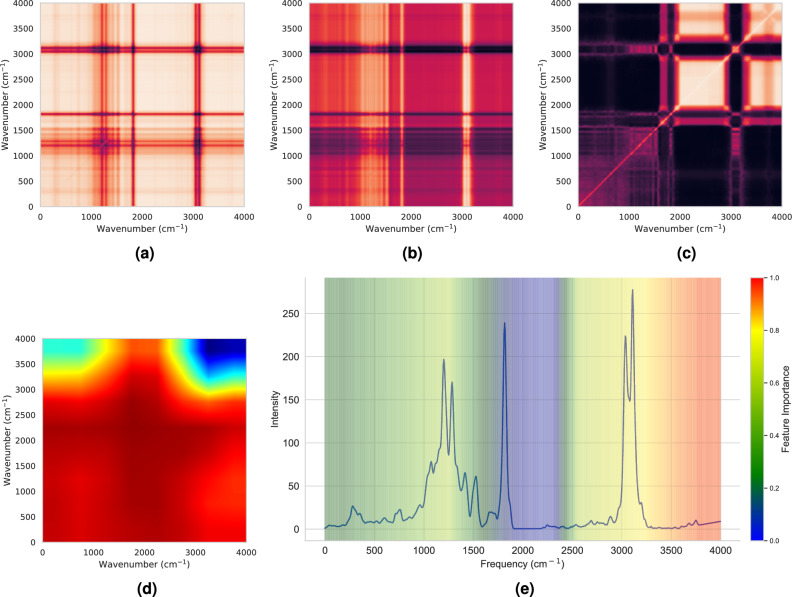
Diagram of saliency analysis of model trained on VM_IMG. (**a**), (**b**), (**c**) and (**d**) represents the average GASF, GADF, MTF, and saliency map of 821 molecules with “fruity” order, each of size 224X224. (**e**) shows the significant signal locations overlayed on an average VS of 821 molecules. In (**a**), (**b**), (**c**), (**d**) and (**e**) red color signifies higher values/importance.

To understand what model has learned from spectra, GradCAM++ was employed to generate saliency maps, revealing the most crucial regions for classification. For the 821 molecules exhibiting the most frequent fruity smell, Fig. 9 displays the average of (a) GASF, (b) GADF, (c) MTF, and (d) saliency map, each of size 224x224. Additionally, (e) contour maps depict the overlay of feature importance on the average spectra of these 821 molecules, with red indicating higher values that gradually decrease from red to blue. The examination of average saliency maps for each molecule was undertaken to address subtle differences present in individual spectra, with a focus on identifying common and significant patterns of interest^56^. In Fig. 9d, the red regions represent the most crucial areas. Moreover, Fig.9e showcases the significant signal locations (where red denotes the highest importance, decreasing to blue) superimposed on the average vibrational spectra (VS) of 821 molecules with a fruity smell. The noteworthy bands between 1000 and 1500 cm^−1^ and 3000–3700 cm^−1^ underscored the importance, while regions of VS lacking or exhibiting less frequent peaks are shaded in blue. The saliency analysis shows that model focuses in the most physically informative regions of VS. This aligns with the intuition that humans may utilize this approach when comparing various spectra. Supplementary file 2 provides saliency maps for the three other most frequent smell categories, namely ‘sweet’, ‘green’, and ‘floral’.

## Conclusion

We compiled a novel and extensive dataset of VS for odorant molecules; prior studies have utilized very few molecules to study the relation between VS and odour^57^. We systematically analyzed them using deep learning-based classification techniques and performed clustering to understand the relation between VS, structure, and complex odor perception. We utilized GASF-GADF-MTF to transform VS into images to improve the classification results further. Furthermore, we try a fusion of VS and traditional DFF features to improve the classification results. A significant limitation of our study lies in the fact that it is currently confined to vibrational spectra (VS) of only 3018 molecules. A more expansive dataset could offer enhanced insights into the relationship between VS and odor. The outcomes of this research fortify the proposition that vibration plays a significant role in olfaction. Moreover, it suggests that the capabilities of biological olfaction might be replicable through vibration-based sensing and identification. The results derived from saliency analysis underscore the specific regions where the model concentrates its attention during decision-making, highlighting the potential of VS in facilitating automated odor classification and artificial odor design for applications such as perfumes and cosmetics. This study also underscores the capacity of deep learning to advance the field of odor classification.

### Supplementary Information

Supplementary Information 1.

Supplementary Information 2.

Supplementary Information 3.

Supplementary Information 4.

Supplementary Information 5.

## Data Availability

The Vibrational Spectra (VS) used in this work were acquired from the Chemistry Webbook hosted by the National Institute of Standards and Technology (NIST)^24^. A list of molecules of IGD and Subset-IGD (Supplementary File 1), along with implementation details of classification models (Supplementary File 2), are provided as supporting information.
