# Supplementary material for: Predicting odor from vibrational spectra: a data-driven approach
Source: Sci Rep. 2024 Sep 2;14:20321. doi: 10.1038/s41598-024-70696-w (PMC11369114; doi:10.1038/s41598-024-70696-w)
Supplement: Supplementary file 2 — Supplementary Information 2. [file 41598_2024_70696_MOESM2_ESM.pdf]

Predicting Odor from Vibrational Spectra: a Data-Driven Approach: SUPPORTING INFORMATION

Durgesh Ameta[1,4], Laxmidhar Behera[1,2], Aniruddha Chakraborty[3], and Tushar Sandhan[2,*]

[1]Indian Knowledge System and Mental Health Applications Centre, Indian Institute of Technology, Mandi, 175005, India
[2]Department of Electrical Engineering, Indian Institute of Technology, Kanpur, 208016, India
[3]School of Basic Sciences, Indian Institute of Technology, Mandi, 175001, India
[4]Indian Knowledge System Centre, ISS, Delhi, 110065, India
[*]sandhan@iitk.ac.in

1 Architecture of models used for classification

As described in **MATERIALS AND METHODS** section of manuscirpt we utilized a combination of weighted binary cross entropy (WBCEL) and focal loss to deal with class imbalance[1–3]. Here we provide more implementation details of models used for classification. All these models are cost-sensitive multilayer perceptron (CSMLP) with fully connected layers, for the activation function, we have used Relu, and at the final layer, we used sigmoid. Models were trained with Adam[4](keras-optimizer), and dropout was used as a regularizer to prevent overfitting.

1.1 CSMLP trained on Subset-IGD (PCA-Reduced VS_IMG concatenated to PCA-Reduced DFF)

Details of the multi-label classifier used in the fusion model.

Layers	Shape	Parameters
dense(Dense)	(None,800)	2712800
dropout(Dropout)	(None,800)	0
dense_1(Dense)	(None,300)	240300
dropout_1(Dropout)	(None,300)	0
dense_2(Dense)	(None,150)	45150
dense_3(Dense)	(None,109)	16459

Supplementary Table I. Details of the multi-label classifier used in the fusion model. Model Layers, Shapes, and Parameters.

Threshold for sigmoid = 0.409;
Loss Function = FL + BCE;
γ =2;
$\varepsilon = e^{-7}$;
δ = 1/4;
epochs =100

'he-uniform' kernel initializer and ReLU activation were applied for all the dense layers except the output layer. For the output layer, sigmoid activation function was applied.

1.2 CSMLP trained on IGD(DFF)

Details of the multi-label classifier trained on DFF of IGD.

**Layers**	**Shape**	**Parameters**
dense(Dense)	(None, 1024)	1049600
dropout(Dropout)	(None, 1024)	0
dense_1(Dense)	(None, 300)	307500
dropout_1(Dropout)	(None, 300)	0
dense_2(Dense)	(None, 150)	45150
dense_3(Dense)	(None, 109)	16459

Supplementary Table II. Details of the multi-label classifier trained on DFF of IGD. Model Layers, Shapes, and Parameters

Threshold for sigmoid = 0.393;
Loss Function = 0.5*FL + BCE;
γ =2;
$\varepsilon = e^{-7}$;
δ = 1/4;
epochs = 100.

'he-uniform' kernel initializer and ReLU activation were applied for all the dense layers except the output layer. For the output layer, sigmoid activation function was applied.

1.3 CSMLP trained on Subset-IGD(GS_VS)

Details of the multi-label classifier trained on GS_VS of Subset-IGD.

Layers	Shape	Parameters
dense(Dense)	(None,800)	640800
dropout(Dropout)	(None,800)	0
dense_1(Dense)	(None,300)	240300
dropout_1(Dropout)	(None,300)	0
dense_2(Dense)	(None,150)	45150
dense_3(Dense)	(None,109)	16459

Supplementary Table III. Details of the multi-label classifier trained on GS_VS of Subset-IGD. Model Layers, Shapes, and Parameters

Threshold for sigmoid = 0.407;
Loss Function = 0.5*FL + BCE
γ =2,
$\varepsilon = e^{-7}$,
δ = 1/4
epochs = 100

'he-uniform' kernel initializer and ReLU activation were applied for all the dense layers except the output layer. For the output layer, sigmoid activation function was applied.

1.4 CSMLP trained on Subset-IGD(DFF)

Details of the multi-label classifier trained on DFF of Subset-IGD.

Layers	Shape	Parameters
dense(Dense)	(None,900)	922500
dropout(Dropout)	(None,900)	0
dense_1(Dense)	(None,300)	270300
dropout_1(Dropout)	(None,300)	0
dense_2(Dense)	(None,150)	45150
dense_3(Dense)	(None,109)	16459

Supplementary Table IV. Details of the multi-label classifier trained on DFF of Subset-IGD. Model Layers, Shapes, and Parameters.

Threshold for sigmoid = 0.412
Loss Function = BCE
δ = 1/4
epochs = 100

'he-uniform' kernel initializer and ReLU activation were applied for all the dense layers except the output layer. For the output layer, sigmoid activation function was applied.

1.5 CSMLP trained on Subset-IGD(VS_IMG)

Details of the multi-label classifier trained on VS_IMG of Subset-IGD.

Layers	Shape	Parameters
dense(Dense)	(None,800)	2429600
dropout(Dropout)	(None,800)	0
dense_1(Dense)	(None,300)	240300
dropout_1(Dropout)	(None,300)	0
dense_2(Dense)	(None,150)	45150
dense_3(Dense)	(None,109)	16459

Supplementary Table V. Details of the multi-label classifier trained on VS_IMG of Subset-IGD. Model Layers, Shapes, and Parameters.

Threshold for sigmoid = 0.407
Loss Function = 0.5*FL + BCE
γ =2,
$\varepsilon = e^{-7}$,
δ = 1/4
epochs = 100

'he-uniform' kernel initializer and ReLU activation were applied for all the dense layers except the output layer. For the output layer, sigmoid activation function was applied.

2 Saliency Analysis with GradCam++

The explainability of the classification model is very important for developing vibration-based biomimetic sensors. We used gradCam++ for saliency analysis of CNN models[5]. GradCam++ is a model-agnostic method and does not require model retraining. GradCam++ provides visual explanations of CNN model predictions in terms of better object localization than the state-of-the-art.

This section describes the model used for saliency analysis and the saliency map for odors "sweet", "green", and "floral".

2.1 Model Architecture

2.2 CSMLP used for saliency analysis

Details of the multi-label classifier used for saliency analysis. Following the resnet50 we used an encoder to reduce the dimension of the "flatten" layer 131072 features to 3000.

Layers	Shape	Parameters
resnet50(Functional)	(None, 8, 8, 2048)	23587712
layer(Layer)	(None, 8, 8, 2048)	0
flatten(Flatten)	(None, 131072)	0
sequential(Sequential)	(None, 3000)	687372000
dense(Dense)	(None, 800)	2400800
dropout(Dropout)	(None, 800)	0
dense(Dense)	(None, 300)	240300
dropout(Dropout)	(None, 300)	0
dense(Dense)	(None, 150)	45150
dense(Dense)	(None, 109)	16459

Supplementary Table VI. Details of the multi-label classifier used for saliency analysis. Model Layers, Shapes, and Parameters.

Threshold for sigmoid = 0.44
Loss Function = BCE
δ = 1/4
epochs = 100

'he-uniform' kernel initializer and ReLU activation were applied for all the dense layers except the output layer. For the output layer, sigmoid activation function was applied.

2.3 Saliency plots

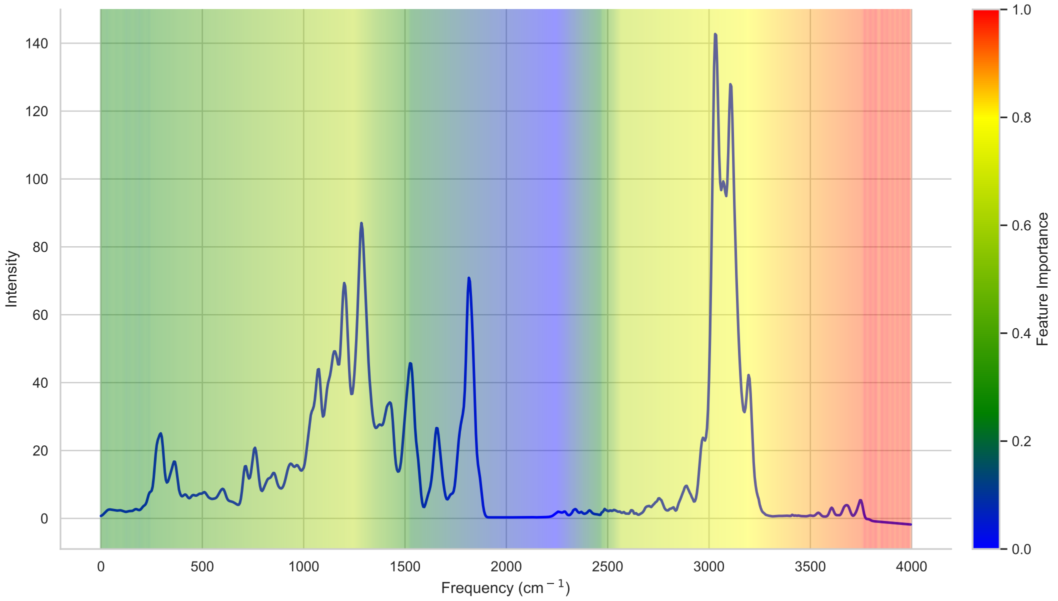

Supplementary Figure I. Diagram of saliency analysis of model trained on VM_IMG for odor "floral". Important features overlayed on average VS of 420 molecules with "floral" odor. The red color signifies higher values/importance.

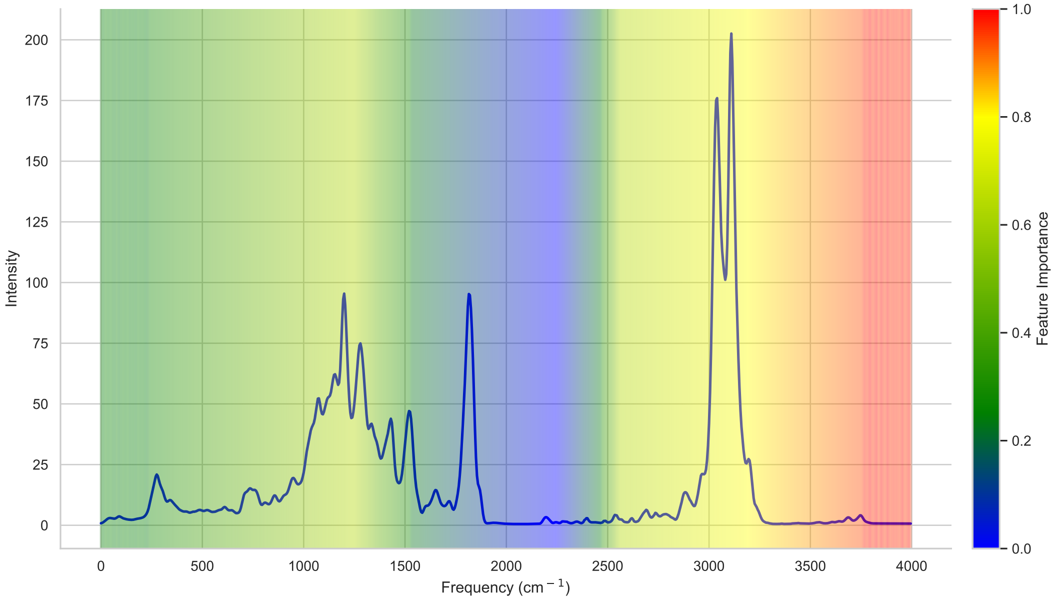

Supplementary Figure II. Diagram of saliency analysis of model trained on VM_IMG for odor "green". Important features overlayed on average VS of 500 molecules with "green" odor. The red color signifies higher values/importance.

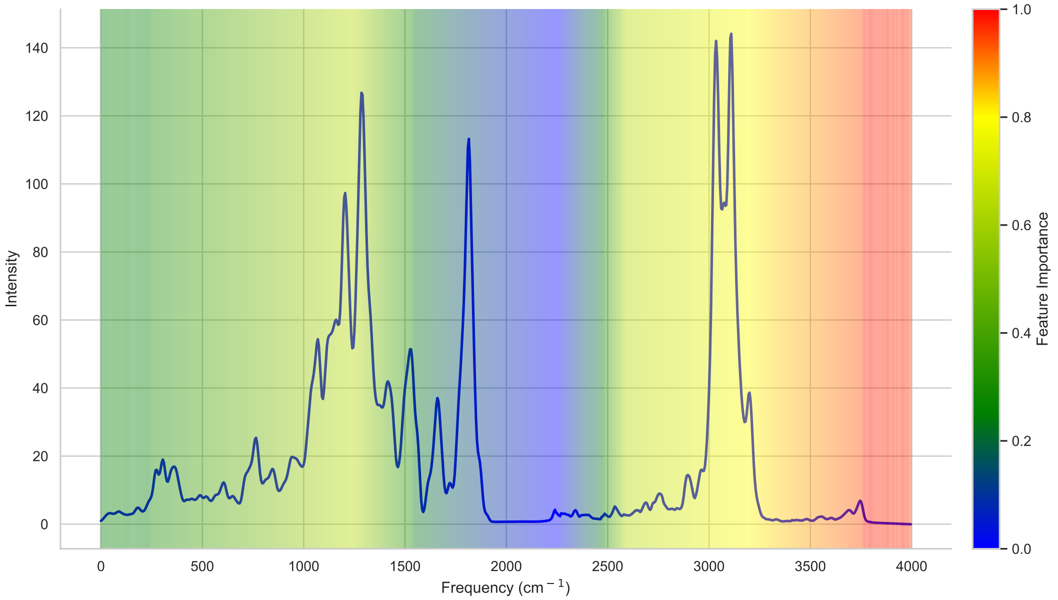

Supplementary Figure III. Diagram of saliency analysis of model trained on VM_IMG for odor "sweet". Important features overlayed on average VS of 552 molecules with "sweet" odor. The red color signifies higher values/importance.

3 Elbow curves

Plots below are elbow curves for two-dimensional GS_VS and VS_IMG. To identify the most suitable number of clusters, we generated an “elbow" curve depicting intra-cluster variability in relation to the number of clusters.

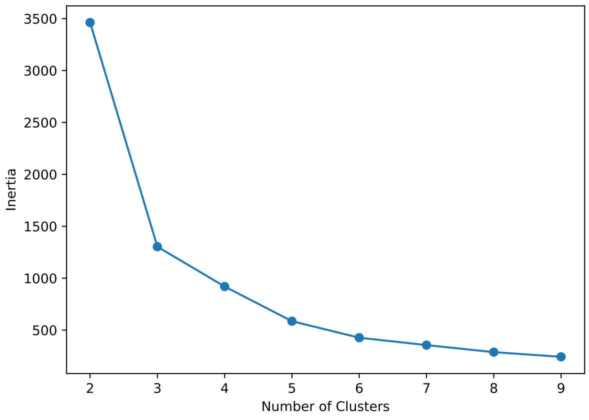

(a) PCA

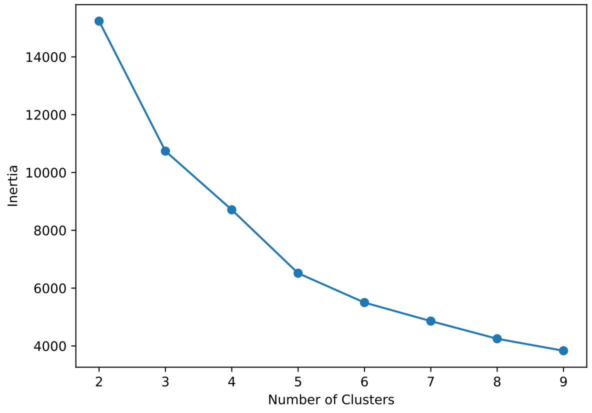

(b) MDS

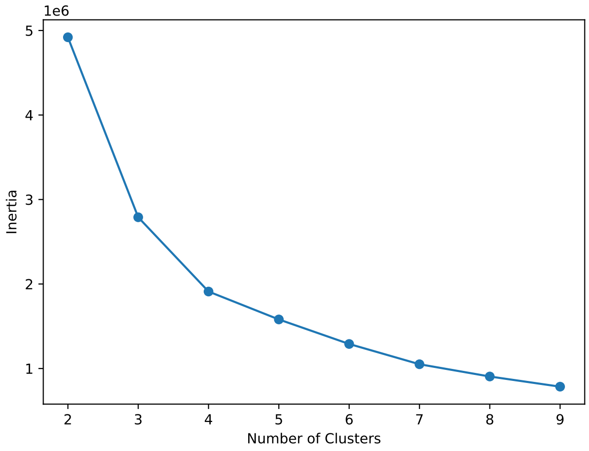

(c) t-SNE

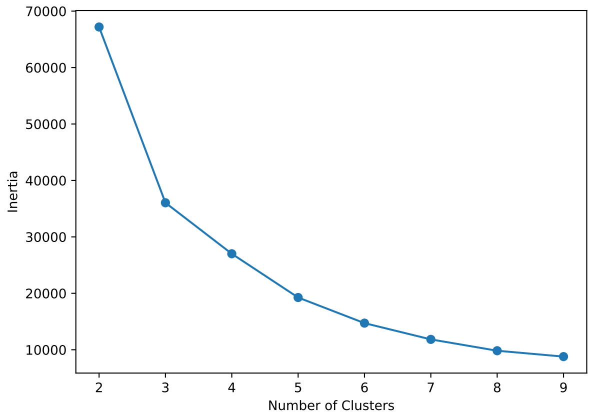

(d) UMAP

Supplementary Figure IV. Elbow curves for two-dimensional GS_VS features reduced using PCA, MDS, t-SNE, UMAP. Representation of intra-cluster variability as a function of the number of clusters. The optimal number of clusters is around the bend of the curve.

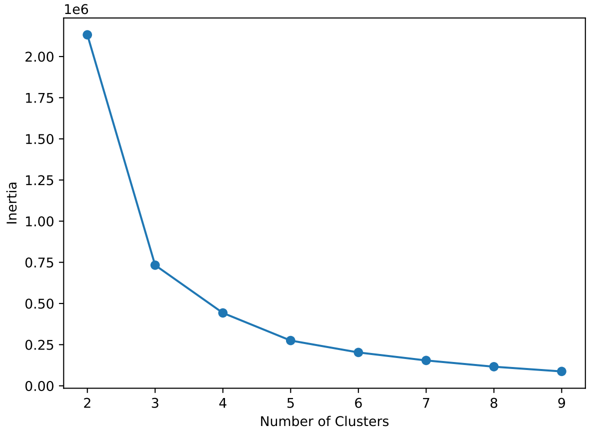

(a) PCA

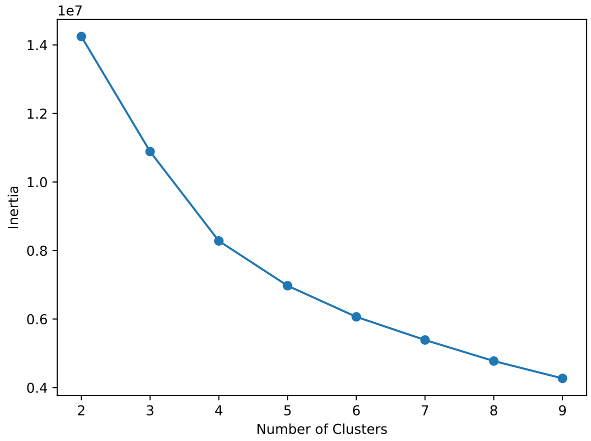

(b) MDS

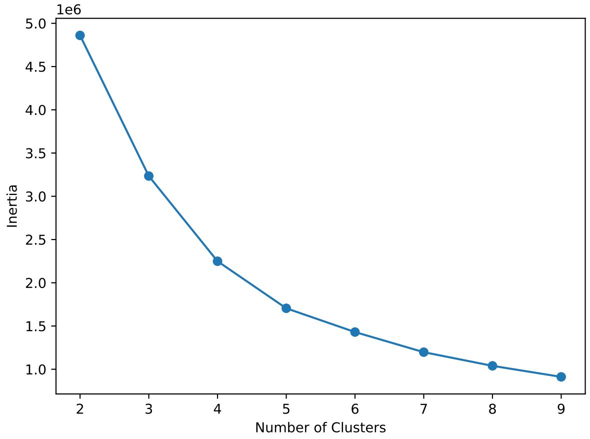

(c) t-SNE

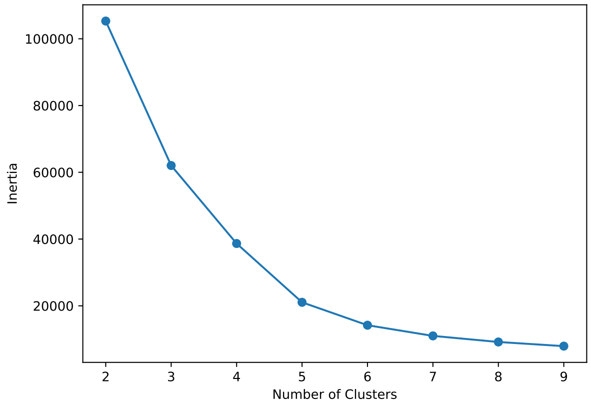

(d) UMAP

Supplementary Figure V. Elbow curves for two-dimensional VS_IMG features reduced using PCA, MDS, t-SNE, UMAP. Representation of intra-cluster variability as a function of the number of clusters. The optimal number of clusters is around the bend of the curve.

References

1. Wang, Y.-X., Ramanan, D. & Hebert, M. Learning to model the tail. In Guyon, I. *et al.* (eds.) *Advances in Neural Information Processing Systems*, vol. 30 (Curran Associates, Inc., 2017).

2. Mikolov, T., Sutskever, I., Chen, K., Corrado, G. & Dean, J. Distributed representations of words and phrases and their compositionality, DOI: 10.48550/ARXIV.1310.4546 (2013).

3. Huang, C., Li, Y., Loy, C. C. & Tang, X. Learning deep representation for imbalanced classification. In *2016 IEEE Conference on Computer Vision and Pattern Recognition (CVPR)*, 5375–5384, DOI: 10.1109/CVPR.2016.580 (2016).

4. Keras Developers. Keras documentation - optimizers (2023). Accessed: 7-12-23.

5. Chattopadhay, A., Sarkar, A., Howlader, P. & Balasubramanian, V. N. Grad-cam++: Generalized gradient-based visual explanations for deep convolutional networks. In *2018 IEEE Winter Conference on Applications of Computer Vision (WACV)*, 839–847, DOI: 10.1109/WACV.2018.00097 (2018).
