# Supplementary material for: Predicting odor from vibrational spectra: a data-driven approach
Source: Sci Rep. 2024 Sep 2;14:20321. doi: 10.1038/s41598-024-70696-w (PMC11369114; doi:10.1038/s41598-024-70696-w)
Supplement: Supplementary file 5 — Supplementary Information 5. [file 41598_2024_70696_MOESM5_ESM.pdf]

Predicting Odor from Vibrational Spectra: a Data-Driven Approach: SUPPORTING INFORMATION

Durgesh Ameta[1,4], Laxmidhar Behera[1,2], Aniruddha Chakraborty[3], and Tushar Sandhan[2,*]

[1] Indian Knowledge System and Mental Health Applications Centre, Indian Institute of Technology, Mandi, 175005, India
[2]Department of Electrical Engineering, Indian Institute of Technology, Kanpur, 208016, India
[3]School of Basic Sciences, Indian Institute of Technology, Mandi, 175001, India
[4]Indian Knowledge System Centre, ISS, Delhi, 110065, India *sandhan@iitk.ac.in

We tried another frequently used molecular descriptor, the Morgan fingerprint, also known as the extended-connectivity fingerprint (ECFP4) [1]. The bit length was set to 2048 bits, the fragment radius was set to 2 (diameter 4), and RDKit was used to generate these features [3]. With cost-sensitive MLP (CSMLP), we classify ECFP4 and features obtained by concatenating Daylight fingerprints and ECFP4 on IGD and Subset-IGD datasets. The results are presented in **Supplementary Table VII** and **Supplementary Table VIII**.

Features	Precision	Recall	F1_score
Daylight fingerprint (1024)	0.3632	0.4614	0.4064
Morgan fingerprint (2048)	0.3285	0.4012	0.3612
Concatenated Morgan and Daylight Features (3072)	0.3222	0.4348	0.3702

Supplementary Table VII. Precision, recall, and **F1 score** on the Subset-IGD dataset for Daylight, Morgan fingerprints, and features obtained after concatenation of both with CSMLP.

Features	Precision	Recall	F1_score
Daylight fingerprint (1024)	0.3603	0.4710	0.4083
Morgan fingerprint (2048)	0.3714	0.4298	0.3985
Concatenated Morgan and Daylight Features (3072)	0.3606	0.4437	0.3978

Supplementary Table VIII. Precision, recall, and **F1 score** on the IGD dataset for Daylight, Morgan fingerprints, and features obtained after concatenation of both with CSMLP.

To address the data imbalance we have tried cost-sensitive MLP in the paper; we also tried Multi-Label Random Under-Sampling (ML-RUS) and Multi-Label Random Over-Sampling (ML-ROS) techniques as augmented sampling techniques.

Resampling methods are often used to address imbalanced data in multi-label datasets (MLDs). These methods involve preprocessing the MLDs by either removing samples from the majority label (undersampling) or creating new samples for the minority label (oversampling) [2]. Sometimes, a combination of both methods is used. Depending on how samples are added or removed, these methods can be categorized as random or heuristic. Random methods choose samples to delete or create randomly, while heuristic methods use specific rules to identify and create the right instances. In this study, we specifically utilize random resampling methods. Random resampling methods for multi-label classification (MLC) can be based on the Label Powerset (LP) transformation, BR methods, imbalance measures, etc. However, LP-based resampling can be limited due to the sparsity of labels in MLDs, where there can be as many unique label combinations as instances, causing all label-sets to be both majority and minority cases simultaneously. An alternative approach to address this issue is focusing on each individual label's imbalance level. Examples of such methods are Multi-Label Random Under-Sampling (ML-RUS) and Multi-Label Random Over-Sampling (ML-ROS), which concentrate on the frequency of individual labels rather than the entire label-sets, isolating instances with one or more minority labels. In this study, we utilize ML-RUS and ML-ROS [2]. We used a label cardinality Card, defined as shown in Eq. (1), and label density Dens, Eq. (2) scores to characterize MLDs and was used during resampling to evaluate the imbalance [4].

$$Card(D) = \sum_{i=1}^{|D|} \frac{|Y_i|}{|D|}. \quad (1)$$

$$Dens(D) = \frac{Card(D)}{|Y|}. \quad (2)$$

The resampling results are presented in **Supplementary Table IX** and **Supplementary Table X** for Subset-IGD and IGD respectively.

**Resampling - %**	**RFC**	**BRC**	**CC**	**CSMLP**
MLRUS-10	0.3144	0.3216	0.3155	0.3928
MLRUS-20	0.3105	0.3241	0.3119	0.3884
MLRUS-30	0.3024	0.3350	0.2913	0.3894
MLROS-10	0.3234	0.3410	0.3399	**0.3982**
MLROS-20	**0.3268**	**0.3466**	0.3394	0.3911
MLROS-30	0.3213	0.3444	**0.3436**	0.3978
**Baseline**	**0.3188**	**0.3428**	**0.3245**	**0.3838**

Supplementary Table IX. This table compares the performance of RF, BR, CC, and CSMLP algorithms in various resampled versions of the Subset-IGD dataset on Daylight Fingerprint features. The evaluation uses Multi-Label Random Under-Sampling (MLRUS) and Multi-Label Random Over-Sampling (MLROS) techniques with sampling ratios of 10%, 20%, and 30%. Micro-averaged F1-scores are reported for each algorithm-dataset pair and compared against the Baseline results obtained from CSMLP on the original dataset.

Resampling - %	RFC	BRC	CC	CSMLP
MLRUS-10	0.3209	0.3422	0.3275	**0.4095**
MLRUS-20	0.3052	0.3337	0.3182	0.3900
MLRUS-30	0.2999	0.3268	0.3067	0.3951
MLROS-10	0.3199	0.3524	0.3291	0.4078
MLROS-20	0.3240	0.3502	**0.3299**	0.4072
MLROS-30	**0.3242**	**0.3527**	0.3262	0.4047
Baseline	0.3190	0.3499	0.3260	0.4061

Supplementary Table X. This table compares the performance of RF, BR, CC, and CSMLP algorithms in various resampled versions of the IGD dataset on Daylight Fingerprint features. The evaluation uses Multi-Label Random Under-Sampling (MLRUS) and Multi-Label Random Over-Sampling (MLROS) techniques with sampling ratios of 10%, 20%, and 30%. Micro-averaged F1-scores are reported for each algorithm-dataset pair and compared against the Baseline results obtained from CSMLP on the original dataset.

References:-

[1] Zhong, S., & Guan, X. (2023). Count-based Morgan fingerprint: A more efficient and interpretable molecular representation in developing machine learning-based predictive regression models for water contaminants' activities and properties. *Environmental Science & Technology*, *57*(46), 18193–18202. https://doi.org/10.1021/acs.est.3c02198

[2] Charte F, Rivera AJ, del Jesus MJ, Herrera F. Addressing imbalance in multilabel classification: Measures and random resampling algorithms. Neurocomputing. 2015;163:3–16. doi:10.1016/j.neucom.2014.08.091

[3] Landrum GA (2018) RDKit: open source cheminformatics. http://www. rdkit.org

[4] M.L. Zhang, Z.H. Zhou, A review on multi-label learning algorithms, IEEE Trans. Knowl. Data Eng. 8 (2014) 1819–1837. http://dx.doi.org/10.1109/TKDE.2013.39
